# Supplementary material for: Ultrathin wide-angle large-area digital 3D holographic display using a non-periodic photon sieve
Source: Nat Commun. 2019 Mar 21;10:1304. doi: 10.1038/s41467-019-09126-9 (PMC6428928; doi:10.1038/s41467-019-09126-9)
Supplement: Supplementary file 1 — Supplementary Information [file 41467_2019_9126_MOESM1_ESM.docx]

**Supplementary Information**

Ultrathin wide-angle large-area digital 3D holographic display using a non-periodic photon sieve

**Jongchan Park^1,2^, KyeoReh Lee^1,2^, and YongKeun Park^1,2,3*^**

^1^Department of Physics, Korea Advanced Institute of Science and Technology, Daejeon 34141, Republic of Korea.

^2^KAIST Institute for Health Science and Technology, KAIST, Daejeon 34141, Republic of Korea.

^3^Tomocube, Inc., Daejeon 34051, Republic of Korea

*Correspondence: Prof. YongKeun Park, Department of Physics, Korea Advanced Institute of Science and Technology, Daejeon 34141, Republic of Korea. Tel: (82) 42-350-2514, Email: [yk.park@kaist.ac.kr](mailto:yk.park@kaist.ac.kr)


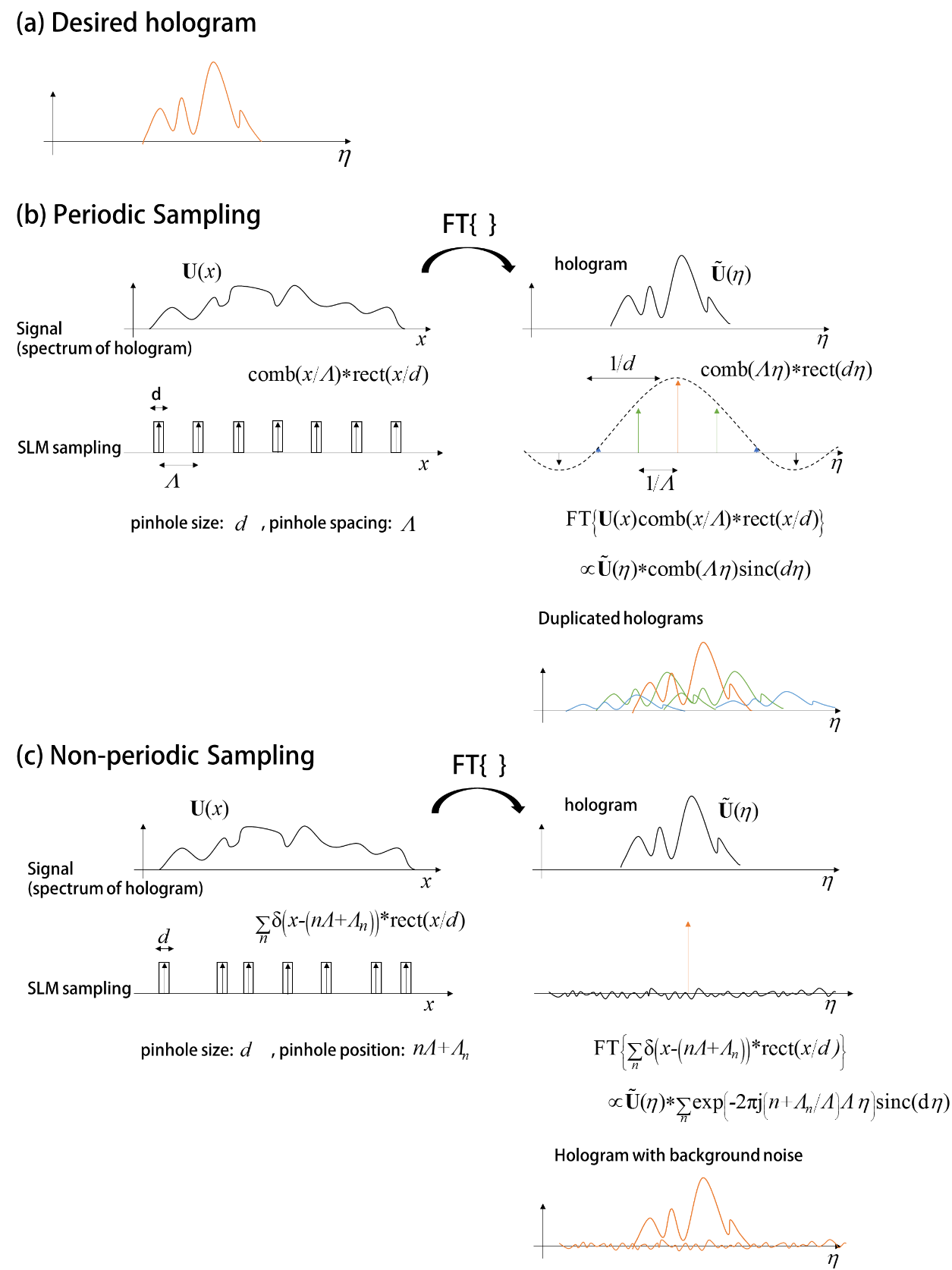


**Supplementary Figure 1 | Comparison between Fourier holograms from periodic photon sieve and non-periodic photon sieve. a**, A hologram of interest. **b**, If the desired hologram contains high spatial frequency components beyond the Nyquist frequency defined by a pixel pitch of a spatial light modulator, a hologram generated from a periodic photon sieve contains undesirable multiple cloned images. Therefore, the size of the hologram is limited by the pixel pitch rather than the size of the pinholes. **c**, A hologram with a non-periodic photon sieve can carry high spatial frequency components beyond the pixel pitch of the spatial light modulator, therefore can generate large-sized holograms.

**Supplementary Note 1 : Spatial aliasing artefact from periodic and non-periodic sampling**

For simplicity, we consider a one-dimensional Fourier hologram (Supplementary Figure 1). A hologram pattern is generated by a computer and displayed on a spatial light modulator. The pattern can be directly found by applying the inverse Fourier transform on a desired holographic imaging pattern: $U(x)=FT^{-1}\left\{\tilde{U}(\eta)\right\}$ where $\tilde{U}(\eta)$ is the desired image and $U(x)$ is the Fourier spectrum of it. We note that both the $\tilde{U}(\eta)$ and $U(x)$ are complex-valued signals and assumed that the spatial light modulator can modulate both the amplitude and phase of the light. To generate the hologram, the Fourier spectrum is displayed by the spatial light modulator and sparsely sampled by the photon sieve which have size $d$ and distance $\Lambda$. Then the resultant field scattered from the photon sieve is given as below:

$$U(x) comb\left(\frac{x}{\Lambda}\right)*rect\left(\frac{x}{d}\right)$$

The reconstruction of the holographic image occurs at the far field or at the focus of a lens that is given as:

$$\text{FT}\left\{\mathbf{U}\text{(}x\text{)} \text{comb}\left(\frac{x}{\Lambda}\right)*\text{rect}\left(\frac{x}{d}\right)\right\}\propto\tilde{\mathbf{U}}\text{(}\eta\text{)}*\text{comb}\left(\Lambda\eta\right) \text{sinc}\left(d\eta\right)$$

The term, $\text{comb(}\Lambda\eta\text{)} \text{sinc(}d\eta\text{)}$, has peak values on every $\eta=M/\Lambda$ where $M$ is an integer. As a result, multiple cloned images are overlapped in the reconstructed hologram (Supplementary Fig. 1b).

When using a non-periodic photon sieve where the positions of the pinholes are given as, $(n+\Lambda_{n}/\Lambda)\Lambda$, the hologram from the photon sieve is given as below:

$$\text{FT}\left\{\mathbf{U}\text{(}x\text{)} \sum_{n} \text{δ}\left(x-\left(n+\frac{\Lambda_{n}}{\Lambda}\right)\Lambda\right)*\text{rect}\left(\frac{x}{d}\right)\right\}\propto\tilde{\mathbf{U}}\text{(}\eta\text{)}*\sum_{n} \exp\left(-2\text{πj}\left(n+\frac{\Lambda_{n}}{\Lambda}\right)\Lambda\eta\right)\text{sinc}\left(d\eta\right)$$

Assume that $\Lambda_{n}/\Lambda$ values, which describe the position of the pinholes, have continuous uniform normal distribution. At $\eta=0$, the term in the equation (3), for all $n$*.* Therefore shows strong constructive interference. In contrast, at $\eta=M/\Lambda$, the second term become

$$\sum_{n} \exp\left(-2\text{πj}\left(\frac{\Lambda_{n}}{\Lambda}\right)M\right)\text{sinc}\left(\frac{dM}{\Lambda}\right)$$

which is a summation of random phasors and serves as a random background noise. Therefore, the non-periodicity of the photon sieve suppress the cloned holographic images at the cost of background noises.


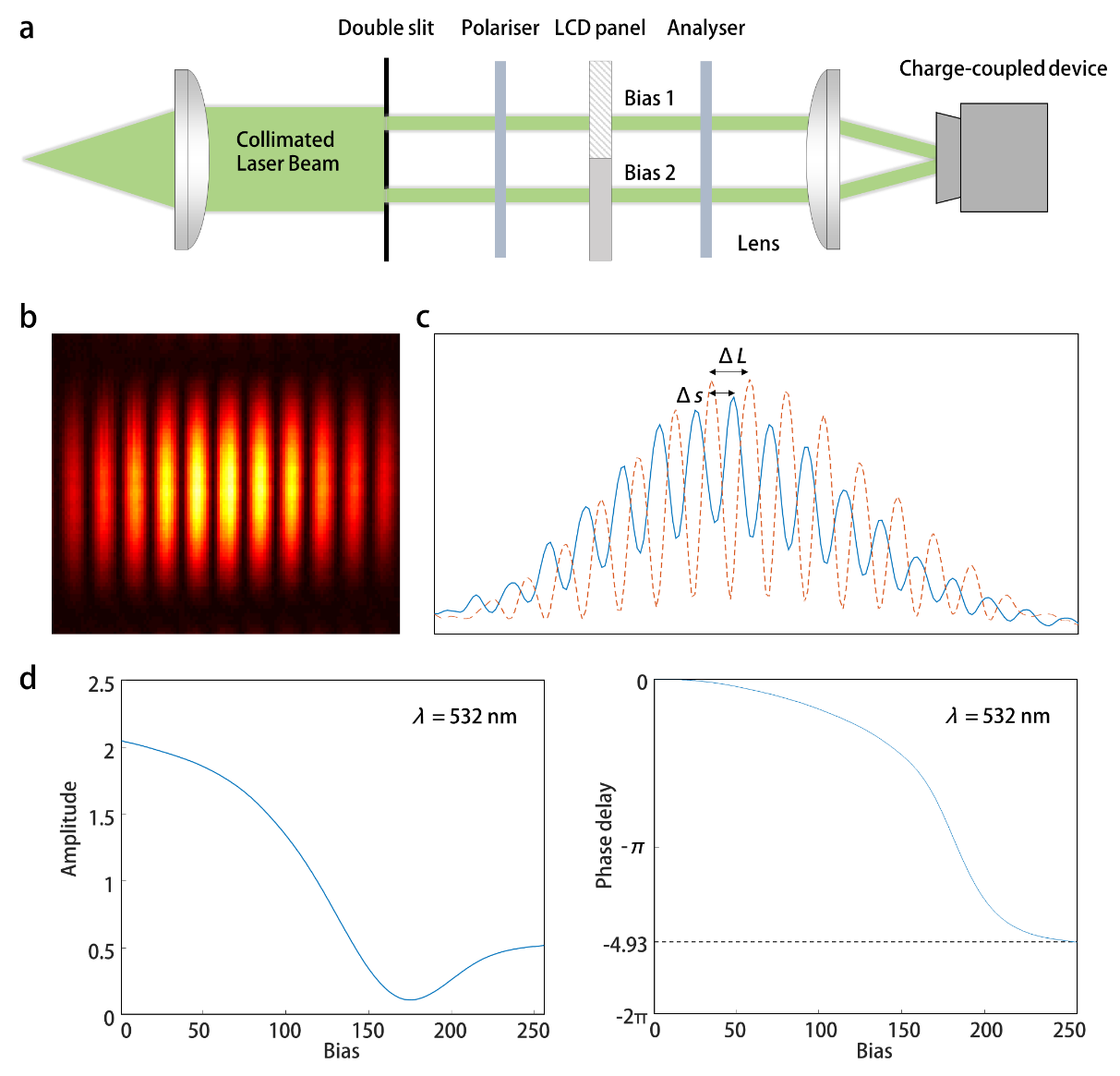


**Supplementary Figure 2 | Double-slit interferometry set-up for characterizing optical response of a transmissive liquid crystal display.** **a**. Optical set-up for measuring phase delay induced by the liquid crystal display (LCD) panel. **b**. Fringe pattern captured by the camera. **c**, The fringe pattern shifts as the bias is applied on the half of the LCD panel. The phase delay is given by: where $\Delta s$ is the later shift of the fringe and $\Delta L$ is the period of the fringe. When measuring the amplitude response of the LCD panel, the double-slit was removed and the total transmittance of the panel corresponding to the applied bias was measured. **d,** Amplitude and phase modulation characteristics of the LCD panel as a function of applied bias at *λ* = 532 nm. A maximum phase delay induced by the LCD panel at given orientations of a polariser and an analyser is 1.57π.

**
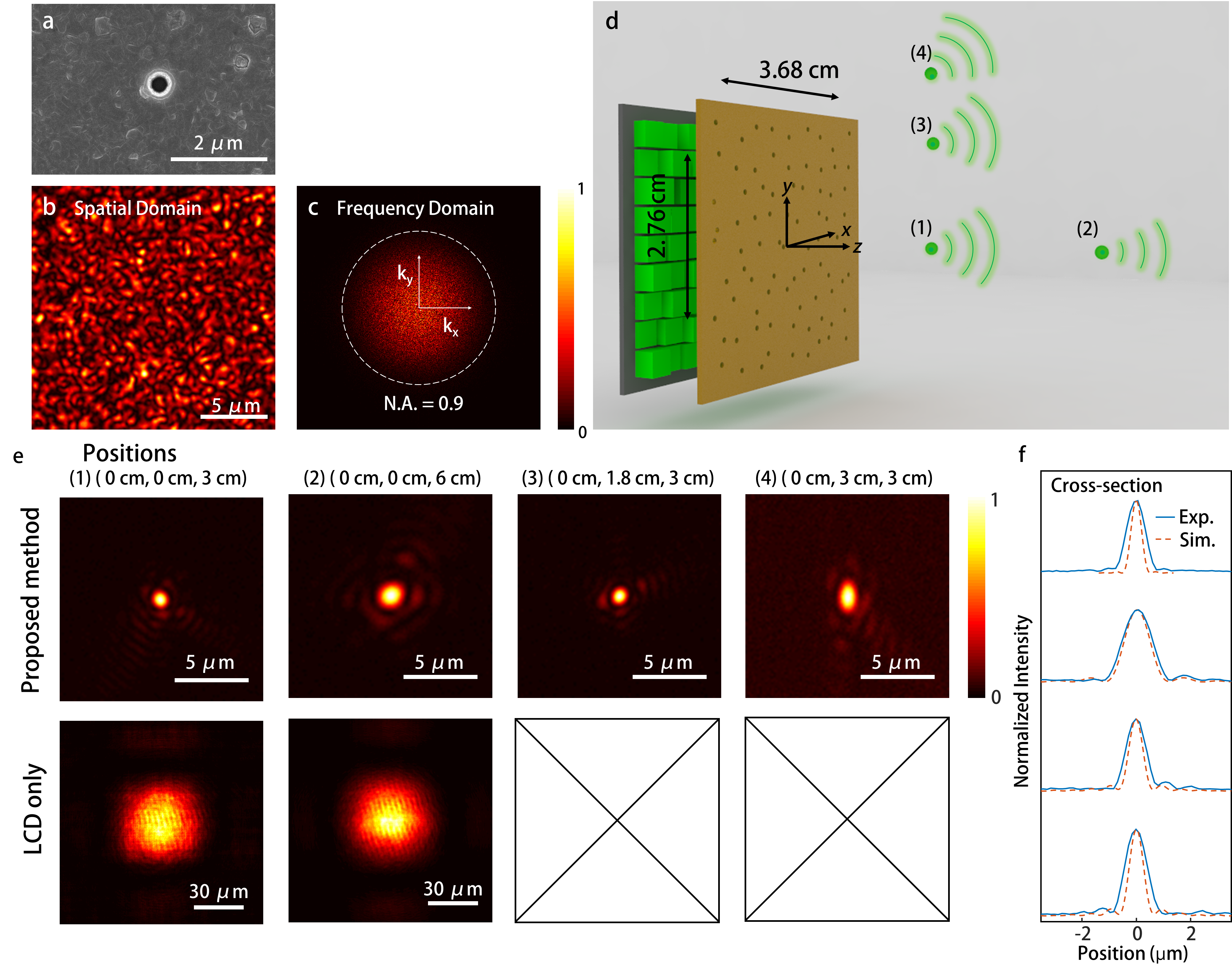
**

**Supplementary Figure 3 | Optical focusing using a non-periodic photon sieve of 400 nm sized pinholes.** **a.** Micrograph from scanning electron microscope of the non-periodic photon sieve fabricated by using a direct e-beam writing method. The sizes of the pinholes in the photon sieve are approximately 400 nm. **b**, Intensity profile of light transmitted through the non-periodic photon sieve. The image was captured using a 4-*f* telescopic imaging system with a high numerical aperture (NA = 0.9) **c**, Spatial frequency map corresponding to the intensity pattern of the image in **b**. **d**, Demonstration of dynamic optical focusing over a wide volume. **e,** Intensity profiles of the optical foci with and without the non-periodic photon sieve. When using the non-periodic photon sieve, the size and shape of the foci are varied because addressable spatial frequency ranges are defined by displaying geometries. In contrast, the sizes of the optical foci are same when focusing at position (1) and (2) using only the LCD panel. In this case, the addressable spatial frequency ranges are limited by a diffraction angle of the LCD panel. The diffraction angle of is given as $\theta=\sin^{-1}(\lambda/2p)$ where $\lambda$ is the wavelength of light and *p* is the pixel pitch of the display. In our demonstration, the diffraction angle is 0.42 degree given the pixel pitch of 36 μm and wavelength of 532 nm. The corresponding numerical aperture is 0.0074 and size (full width at half maximum) of the resultant focus is 37 μm. Due to the limited diffraction angle, no focus is formed at the position (3) and (4) without the photon sieve. **f**. Vertical cross-sections of the intensity profiles in **e**. The lateral sizes (blue solid line) of the foci are compared with the theoretical values (red dashed line) defined by the geometric positions of the foci. The slight broadening of the focus size compared to the theoretically achievable size is a result of the non-uniform diffraction of light field from the pinholes and imperfect correction of aberrations.


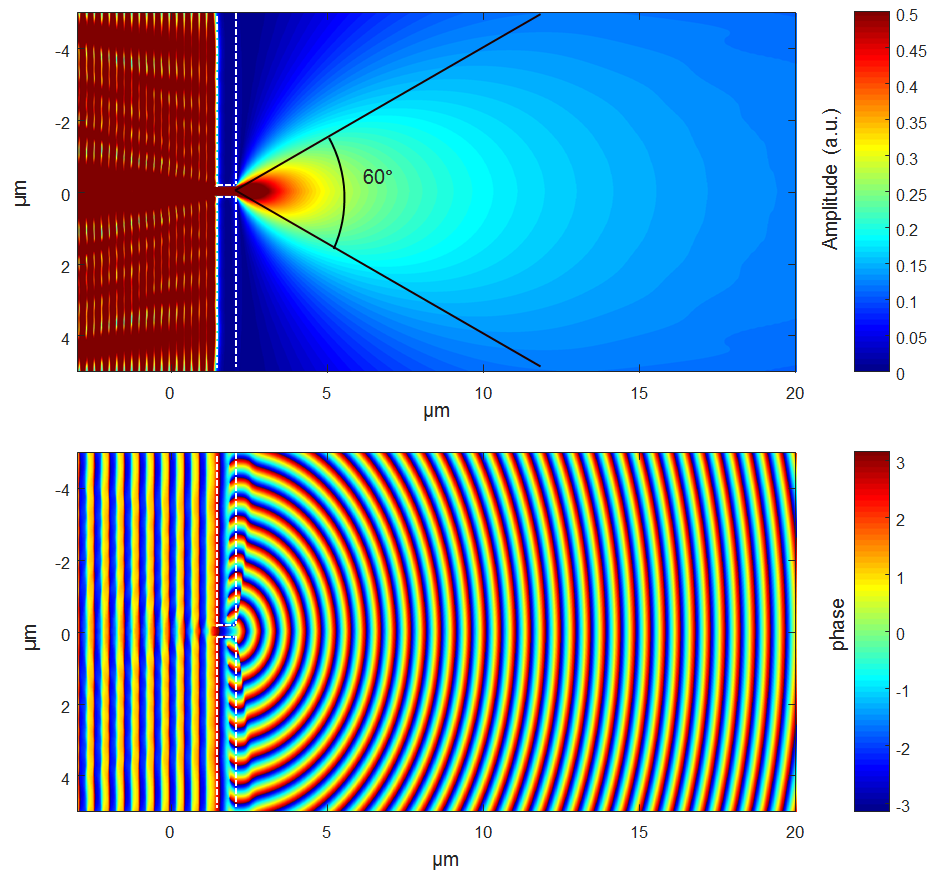


**Supplementary Figure 4 | A FDTD simulation of a diffracting optical field from a 400 nm sized pinhole.** A Far-field intensity and phase maps of the diffracting optical field via the pinhole of 400 nm radius through a metal film of 300 nm was obtained by the FDTD simulation. The incident field is a linearly polarised laser beam with a wavelength of 532 nm. Its large diffraction angle supports large viewing angle of the holographic displays.


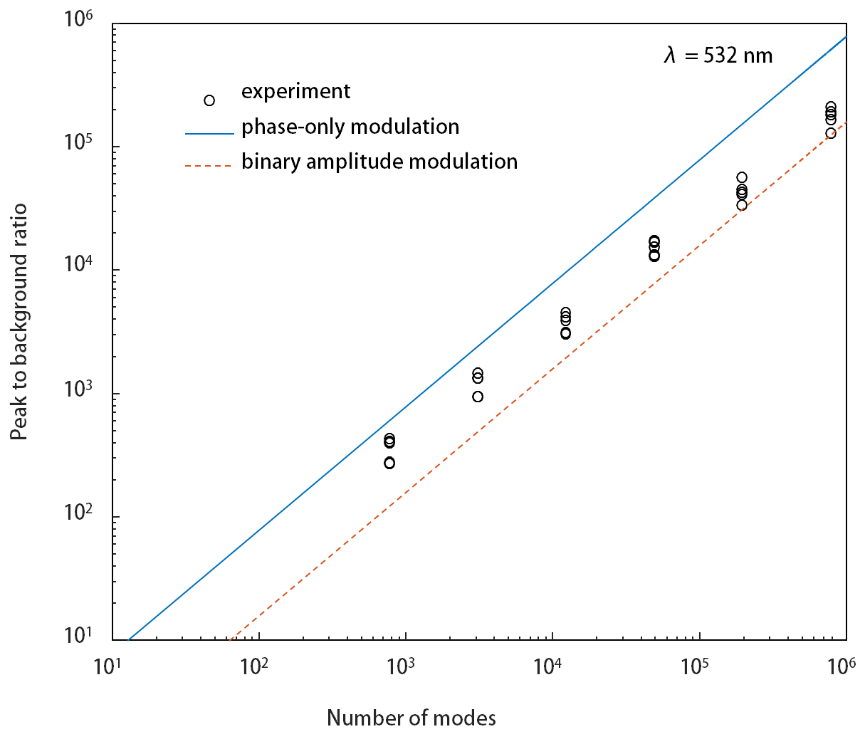


**Supplementary Figure 5 | Signal to background noise ratio of a holographic focus as a function of the number of controlled optical modes.** The ratio between the intensity of the focus and the average value of the background noise was measured as a function of the number of controlled optical modes. The optical response of the LCD panel is shown in Supplementary Figure 2. The wavelength was 532 nm. The maximum value of the experimentally achieved factor is 211,400. For comparison, theoretically achievable values of using phase-only modulation and binary amplitude modulation of spatial light modulators are presented; the slopes are $\pi/4$ and $1/2\pi$, respectively. The theoretical values were achieved under assumption that the entries of the optical transfer functions (or a transmission matrix) of the photon sieve, which connect the pinholes to the focusing position, are statistically independent and obey a circular Gaussian distribution[^1^](#_ENREF_1),[^2^](#_ENREF_2).


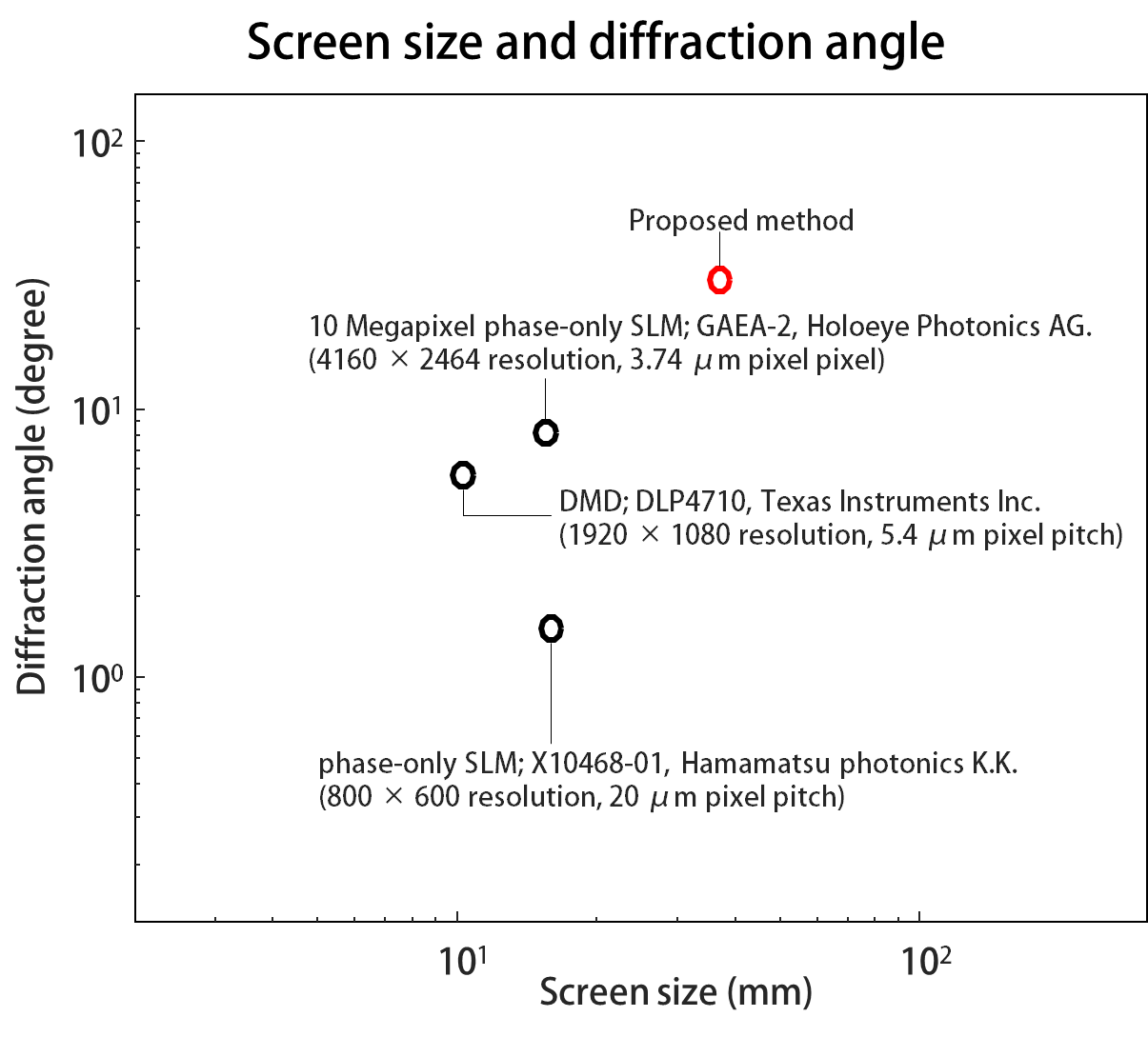


**Supplementary Figure 6 | Comparison between the proposed method and commercial spatial light modulators.** The diffraction angle and screen size of the holographic display with non-periodic photon sieve are denoted by the red circle. The characteristics of the commercially available spatial light modulators are also presented for comparison.

**Supplementary References**

1 Vellekoop, I. M. & Mosk, A. Focusing coherent light through opaque strongly scattering media. *Opt. Lett.* **32**, 2309-2311 (2007).

2 Akbulut, D., Huisman, T. J., van Putten, E. G., Vos, W. L. & Mosk, A. P. Focusing light through random photonic media by binary amplitude modulation. *Opt. Express* **19**, 4017-4029 (2011).
