# Supplementary material for: Ultrathin wide-angle large-area digital 3D holographic display using a non-periodic photon sieve
Source: Nat Commun. 2019 Mar 21;10:1304. doi: 10.1038/s41467-019-09126-9 (PMC6428928; doi:10.1038/s41467-019-09126-9)
Supplement: Supplementary file 3 — Description of Additional Supplementary Files [file 41467_2019_9126_MOESM3_ESM.pdf]

Description of Additional Supplementary Files

File Name: Supplementary Movie 1

Description: Dynamic holographic rotating cube with red, green, and blue colours. A total of 360 frames were captured and displayed at a frame rate of 30 Hz. A monochrome charge-coupled device with spectral filters was used to capture each colour frame. The frames were subsequently combined using the CIE 1931 table with normalized luminance.
